# Toll-Like Receptor 4 Signalling and Its Impact on Platelet Function, Thrombosis, and Haemostasis

**DOI:** 10.1155/2017/9605894

**Published:** 2017-10-17

**Authors:** Thomas M. Vallance, Marie-Theres Zeuner, Harry F. Williams, Darius Widera, Sakthivel Vaiyapuri

**Affiliations:** School of Pharmacy, University of Reading, Reading RG6 6UB, UK

## Abstract

Platelets are anucleated blood cells that participate in a wide range of physiological and pathological functions. Their major role is mediating haemostasis and thrombosis. In addition to these classic functions, platelets have emerged as important players in the innate immune system. In particular, they interact with leukocytes, secrete pro- and anti-inflammatory factors, and express a wide range of inflammatory receptors including Toll-like receptors (TLRs), for example, Toll-like receptor 4 (TLR4). TLR4, which is the most extensively studied TLR in nucleated cells, recognises lipopolysaccharides (LPS) that are compounds of the outer surface of Gram-negative bacteria. Unlike other TLRs, TLR4 is able to signal through both the MyD88-dependent and MyD88-independent signalling pathways. Notably, despite both pathways culminating in the activation of transcription factors, TLR4 has a prominent functional impact on platelet activity, haemostasis, and thrombosis. In this review, we summarise the current knowledge on TLR4 signalling in platelets, critically discuss its impact on platelet function, and highlight the open questions in this area.

## 1. Introduction

Platelets are small, anucleated, and short-lived blood cells with a range of important functions beyond their classical roles in haemostasis [1–3]. The function of platelets in haemostasis has been well documented and is linked to their capacity to respond to the damaged endothelium [4–6]. Following vessel damage and initial activation, platelets secrete a wide variety of small molecules and proteins from intracellular granules in order to activate and recruit more circulating platelets and immune cells, such as leukocytes [4]. In addition to these secretion events, platelets undergo dramatic shape changes that enable them to cover the site of injury and prevent bleeding [4]. Thrombosis (blood clot formation) mediated by platelets occurs in the arteries under pathological conditions and significantly obstructs the blood flow to major organs such as the heart and brain resulting in heart attacks and strokes, respectively [7]. In addition to their physiological functions, platelets can be involved in different pathological conditions, for example, in atherosclerosis [8, 9]. If the atherosclerotic plaque ruptures, the exposure of the subendothelial matrix and release of procoagulatory matrix proteins, such as collagen, are sufficient to initiate the formation of a thrombus (blood clot) at this site [4, 10]. Thrombus poses a significant systemic risk because it is formed in a narrowed blood vessel and so has the potential to completely occlude the vessel and trigger a myocardial infarction or ischaemic stroke [10].

Platelets also have pivotal roles in the innate immune system, which includes cells that combat general infections (e.g., neutrophils), and is responsible for the eradication of pathogens to protect the body from infection [11, 12]. During the immune response, platelets have been shown to interact with and respond to many species of Gram-positive and Gram-negative bacteria through different receptors [13, 14]. Moreover, platelets are capable of internalising specific types of bacteria and viruses although the function of this phenomenon is poorly understood [15, 16]. The ability of platelets to participate in such a wide range of functions and their ability to synthesise certain new proteins despite lacking a nucleus have generated significant scientific interest [2, 3, 17].

In addition, platelets play a role in the development of disseminated intravascular coagulation (DIC), a common complication observed in patients with sepsis [18–20]. During DIC, platelets are activated and form smaller thrombi in the microvasculature or aggregates that are sequestered in organs such as the lungs. Together, this leads to thrombocytopenia, a reduction in the number of circulating platelets. Mild thrombocytopenia is defined as less than 1.5 × 10^11^ platelets per litre of blood compared to between 1.5 and 4.0 × 10^11^ in healthy individuals, but more severe thrombocytopenia is defined as less than 0.5 × 10^11^ platelets per litre [6, 20, 21]. Furthermore, it has been discovered that platelets can promote the formation of neutrophil extracellular traps (NETs) which have cytotoxic actions on host cells beyond their beneficial antibacterial effects [22].

Notably, conditions such as sepsis and DIC have been suggested to be linked to several platelet receptors, especially Toll-like receptor (TLR) 4 [8, 18, 22, 23]. In human nucleated cells, especially in professional antigen-presenting cells, the binding of a ligand to TLR1, 2, 4, 5, 6, 7, 8, 9, and 10 results in the activation of the so called myeloid differentiation factor-88- (MyD88-) dependent pathway, whereas TLR3 activates the MyD88-independent pathway [12, 23, 24]. In contrast to most TLRs which signal exclusively through one of the two pathways, TLR4 is able to activate both MyD88-dependent and MyD88-independent signalling [12, 24].

Platelets contain all of the proteins (e.g., MyD88 and interferon regulatory factor 3 (IRF3)) that are required for signal transduction through TLR4 and so at first glance it would appear that platelets utilise the same mechanisms as in nucleated cells [2, 25]. However, as we will explain in more detail in the subsequent sections, this cannot be the case as both the MyD88-dependent and the MyD88-independent pathways culminate in the activation and nuclear translocation of transcription factors, and this step would not be applicable in anucleated cells like platelets [2, 12, 26]. Before examining the evidence for the TLR4 signalling pathways in platelets, it is worth reviewing the pathways in nucleated cells for use as a benchmark.

## 2. TLR4 Signalling in Nucleated Cells

### 2.1. TLR4 Ligands

Lipopolysaccharide (LPS) is a component of Gram-negative bacterial cell membranes and a powerful ligand for TLR4 [27, 28]. LPS is composed of a lipid A moiety (responsible for the molecule's interactions with TLR4), the core oligosaccharide, and the O-antigen polysaccharide [27, 29, 30]. The lipid A moiety is localised in the outer cell membrane and is formed from a 1,4-bis-phosphorylated diglucosamine molecule linked to variable acyl chains (e.g., six chains in *Escherichia coli* LPS) [27, 29]. The phosphate groups and acyl chains of LPS are important for interactions with TLR4, and alterations in these can shift the molecule from being an agonist to an antagonist [27, 31]. LPS may not be the only ligand for TLR4 as damage-associated molecular patterns (DAMPs), such as high-mobility group box 1 (HMGB1) and heat shock proteins (HSPs), have also been suggested to be capable of inducing activation through this receptor [12, 32].

Although the immunogenic region of LPS is inside the bacterial cell membrane, it is capable of eliciting an immune response due to the presence of lipopolysaccharide-binding protein (LBP) [27, 29, 33]. LBP is a soluble protein that is synthesised by hepatocytes and found in the blood [28, 33]. It is capable of binding to areas rich in LPS (e.g., LPS aggregates and Gram-negative bacterial membranes) and promotes the exposure of the molecule's hydrophobic regions [34]. Subsequent to this, LPS monomers, via a process facilitated by albumin, can associate with CD14 (cluster of differentiation 14), a high affinity, horse shoe-shaped, glycosylphosphatidylinositol- (GPI-) anchored membrane protein [28, 31, 33–35]. CD14 forms a dimer with the dimerisation interface at the C-terminal end and LPS-binding pockets at the N-terminal end [33]. The transfer of LPS to TLR4 and the breakdown of LPS aggregates (micelles) into monomers are mediated by CD14 [28, 31, 33, 36]. Albumin can bind LPS, and other hydrophobic molecules, via hydrophobic interactions between domain III (on albumin) and the fatty acid chains of LPS [34]. Furthermore, albumin is capable of transferring LPS to TLR4 on its own although this requires approximately 10-fold higher concentrations of LPS compared to CD14 [34].

### 2.2. TLR4 Receptor

Similarly to CD14 (the molecule responsible for transferring LPS to TLR4), the ectodomains of TLR4 are horse shoe-shaped due to the presence of several leucine-rich repeats (LRRs) [33, 37]. Like other type I membrane-spanning proteins, the membrane-spanning domain of TLR4 is comprised of a single helix that serves to link the intracellular and extracellular domains [31]. The intracellular domain of TLRs contains a Toll/IL-1 receptor (TIR) domain common to all of the adaptor protein molecules involved at this stage of signalling [1, 31].

**Figure 1:**
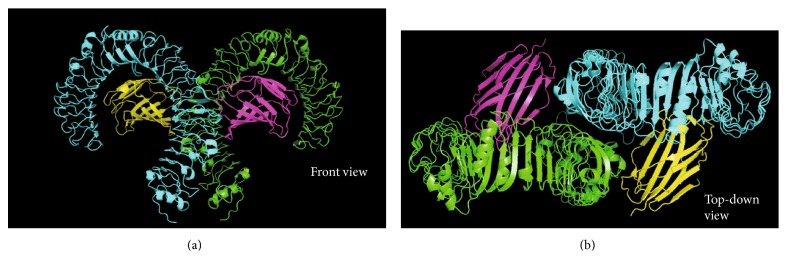
Structure of TLR4/MD-2 ectodomains, in a heterotetrameric form, as seen from (a) or (b). The TLR4 molecule (green) is constitutively bound to MD-2 (magenta), and the TLR4^∗^ (cyan) molecule is constitutively bound to MD-2^∗^ (yellow). Dimerisation interfaces form between TLR4 and MD-2^∗^ and vice versa. Images were created by adapting the structure of TLR4 (PDB code: 3FXI) using PyMOL [37].

For signalling via TLR4 to occur, TLR4 requires heteromeric association with myeloid differentiation factor 2 (MD-2) [38, 39] (Figure 1). MD-2 is required because TLR4 does not bind LPS directly [27]. This is exemplified by the ability of human MD-2 to bind LPS in the absence of TLR4 [31]. MD-2 is constitutively associated with TLR4 through an interaction in the central region of TLR4 and may be responsible for the recognition of different LPS chemotypes [33, 39].

TLR4 has been detected on the plasma membrane and in intracellular compartments (such as the early endosome) of both nucleated cells and platelets [24, 40, 41]. In addition, TLR4 is capable of internalisation, as has been shown following prolonged exposure to LPS [24, 40–42]. The mechanisms behind the internalisation of TLR4 differ between cell types and may be required for MyD88-independent signalling [40, 43]. The intracellular forms of TLRs are not inactive as may be expected for an internalised extracellular receptor but are capable of recognising ligands (such as LPS) in endosomes, lysosomes, and endolysosomes [12, 43, 44]. Notably, plasma membrane localisation of TLR4 requires HSP 90 kDa *β* member 1 (gp96) and protein associated with TLR4 (PRAT4A) acting as chaperones [44, 45]. Moreover, MD-2 has been reported to play a role in TLR4 localisation at the plasma membrane as its absence traps TLR4 in the Golgi apparatus [38].

### 2.3. TLR4 Activation

In order to activate the TLR4 signalling pathway, two receptor complexes need to dimerise to bring together the intracellular TIR domains (Figure 1) [31]. LPS and MD-2 (constitutively bound) binding to TLR4 is required for the TLR4 complex dimerisation to take place [27]. This dimerisation occurs due to the formation of a dimerisation domain that incorporates a hydrophobic patch on TLR4 and one of the acyl chains of LPS [33]. The remaining acyl chains are hidden in the hydrophobic cavity of MD-2 [27]. Ectodomain dimerisation leads to an interaction between the two intracellular domains of the TLR4 monomers [31]. This builds a platform onto which the intracellular signalling complexes can be formed [33]. At this stage, the two pathways diverge but there is still disagreement about what happens during this step [31].

### 2.4. The MyD88-Dependent Pathway

For the MyD88-dependent pathway, the TIR domain-containing adaptor protein (TIRAP), also known as MyD88 adaptor-like (Mal) protein, interacts with the TIR domain of the receptor enabling it to recruit MyD88 to the plasma membrane [31]. TIRAP presence at the plasma membrane is mediated by its phosphatidylinositol-4,5-bisphosphate- (PI(4,5)P_2_-) binding domain [43, 46]. It has been suggested that TIRAP may bind to TLR4's TIR domain using complementary charge distributions because of the observation that charges differ between TLR3 (cannot bind TIRAP) and TLR4 and TLR2 (can or only bind TIRAP, resp.). However, the exact details and structures involved during this binding have not been determined, partially due to the lack of a crystal structure for the TIR domain of TLR4 [31]. Once TIRAP has bound to the receptor, it recruits MyD88 via an interaction between their respective TIR domains [43, 46, 47].

**Figure 2:**
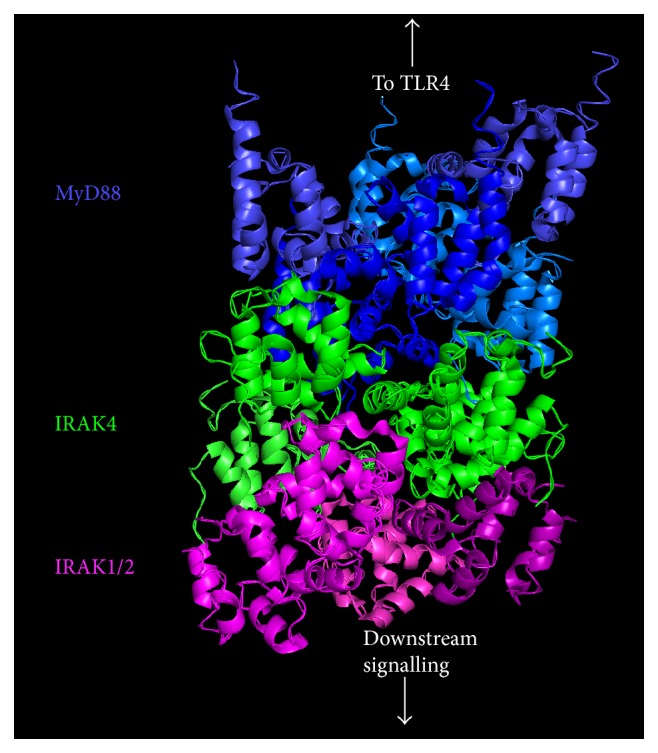
Structure of the Myddosome showing the protein death domains (DD). The Myddosome is formed of six MyD88 molecules, four IRAK4 molecules, and four IRAK1/2 molecules arranged in a single-stranded helix. MyD88 occupies the two layers closest to the plasma membrane whereas IRAK4 and IRAK1/2 form the two subsequent layers. The image was created by adapting the structure of the Myddosome (PDB code: 3MOP) using PyMOL [47].

MyD88 contains a death domain (DD) at its N-terminal end, which is crucial for the subsequent signalling cascade as it enables the construction of a large multimeric complex called the Myddosome (Figure 2) [47]. The Myddosome is formed of six MyD88, four interleukin- (IL-) 1 receptor-associated kinase 4 (IRAK4), and four IRAK1/2 molecules, all of which contain DDs, arranged in a single-stranded left-handed helix [24, 47]. As shown in Figure 2, this helix has multiple levels with the first two levels comprised solely of MyD88, IRAK4 is found in the third level, and IRAK1/2 is found in the fourth level [47]. Following assembly, IRAK4 undergoes an activating autophosphorylation process thereby enabling it to phosphorylate, and activate, IRAK1/2 [47]. Phosphorylation of IRAK1/2 stimulates disassociation from the Myddosome and triggers polyubiquitination of tumour necrosis factor (TNF) receptor-associated factor (TRAF) 6 [47, 48]. TRAF6 interacts with TRAF-activated kinase 1 (TAK) and IRAK1/2, and this complex in turn interacts with NF-*κ*B essential modulator (NEMO) to stimulate the activating phosphorylation of I*κ*B kinase- (IKK-) *β* and the degradation of I*κ*B [24, 49–51]. Degradation of I*κ*B and the release of inhibition on NF-*κ*B permit it to translocate into the nucleus and enhance expression of proinflammatory cytokines including TNF*α* and IL-1*β* [44, 50, 51]. The MyD88-dependent signalling downstream of LPS stimulation is dependent on TLR4 remaining at the plasma membrane as inhibition of internalisation increases NF-*κ*B activity [52].

**Figure 3:**
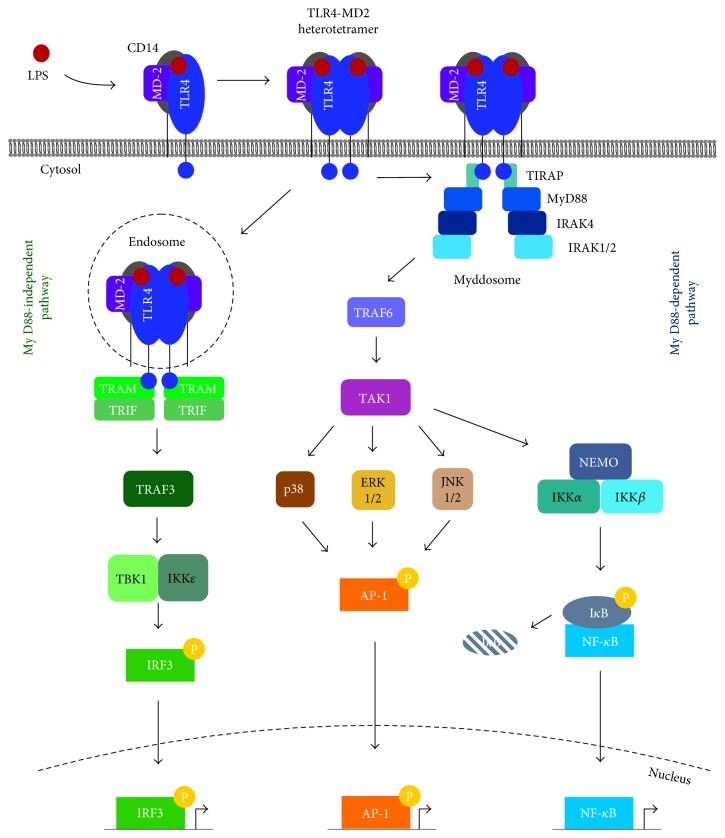
Summary of intracellular TLR4 signalling pathways in nucleated cells. LPS is transferred to CD14 (or albumin), via a process involving LBP and albumin, which transfers LPS to TLR4:MD-2 to complete the heterotetramerisation. There are two major signalling pathways, namely, the MyD88-dependent and -independent pathways, for TLR4 signalling. In the MyD88-dependent pathway, TIRAP (or Mal) enables MyD88 binding to TLR4 and formation of the Myddosome, which contains MyD88, IRAK4, and IRAK1/2. The kinases found at the base of the Myddosome activate TRAF6 and TAK1 followed by the activation of NEMO and its associated kinases. IKK*β* stimulates the degradation of inhibitory I*κ*B, which leads to nuclear translocation of NF-*κ*B and transcription of proinflammatory genes. In addition, TAK1 activates JNK1/2, ERK1/2, and p38, which can then stimulate the transcription factor AP-1. In the MyD88-independent pathway, following CD14-dependent internalisation into the endosomes, TRAM and TRIF are recruited to TLR4 before activating TRAF3. Activation of TRAF3 activates TBK1 and IKK*ε*, which phosphorylate and activate the transcription factor IRF3 that stimulates the transcription of anti-inflammatory cytokines.

Activation of mitogen-activated protein kinases (MAPKs) downstream of MyD88 and TAK1 is also involved in TLR4-mediated responses in nucleated cells [44, 48]. MAPKs include a range of proteins including extracellular signal-regulated kinase (ERK) 1 and 2, c-Jun N-terminal kinase (JNK) 1 and 2, and p38 [48]. These kinases are capable of activating the transcription factor, activator protein 1 (AP-1) [48]. This part of the MyD88-dependent pathway is dependent on the downregulation of TRAF3, via ubiquitination by cellular inhibitor of apoptosis (cIAP), near the plasma membrane where it has a negative regulatory role [48]. A summary of all the signalling pathways in nucleated cells is shown in Figure 3().

### 2.5. The MyD88-Independent Pathway

TRIF-related adaptor molecule (TRAM) is responsible for recruiting TIR domain-containing adaptor-inducing interferon-*β* (TRIF) in the MyD88-independent pathway [31]. Signalling through this pathway occurs following specific internalisation of the TLR4-MD-2 heterotetramer, its bound ligand, and CD14 [53–55]. The protein responsible for the internalisation (clathrin or caveolin) of TLR4 varies between cell types and with time although dynamin and CD14 are always necessary [40, 43, 52, 55]. Whereas CD14 is only required at low concentrations of LPS for MyD88-dependent pathway signalling (with other proteins such as albumin capable of transferring LPS to MD-2), CD14 is always necessary for MyD88-independent signalling [34, 55, 56]. As internalisation of TLR4 occurs, the decrease in PI(4,5)P_2_ in the local area leads to a weakening of the interaction between TLR4 and TIRAP and thus propagates the break-down of the Myddosome [24, 43]. Interestingly, endocytosis of TLR4 does not appear to be dependent on TLR4-mediated signalling, with cells lacking TIRAP, MyD88, TRAM, or TRIF retaining the capacity to internalise the receptor [55]. This has been suggested to be a result of phospholipase C*γ*2 (PLC*γ*2) and spleen-associated tyrosine kinase (Syk) activation in a CD14-dependent and TLR4-independent manner [55].

Upon internalisation, TLR4 enters the endosome, a region of the cell where TRAM and TRAF3 are present and from where MyD88-independent signalling can begin [24, 43, 48, 55]. When recruited to the TLR4-TRAM-TRIF complex by TRIF, TRAF3 is polyubiquitinated thus stimulating the activation of TRAF family member-associated NF-*κ*B activator- (TANK-) binding kinase- (TBK-) 1 and IKK*ε* [48]. TBK1 and IKK*ε* are then free to phosphorylate IRF3, which is activated upon phosphorylation and dimerisation and stimulates the production of type I interferons [48, 56].

## 3. TLR4 Signalling in Platelets

### 3.1. Platelet Activation upon Vascular Damage

The response of platelets to “classical” agonists and the subsequent activation in haemostasis have been well defined [4–6]. During vascular injury, there is exposure of the subendothelial matrix and proaggregatory proteins, such as von Willebrand factor (vWF) and collagen, to the flow of blood. vWF is immobilised on collagen, and its association with GPIb-V-IX, a large glycoprotein (GP) complex, represents the initial interaction between platelets and the damaged vessel. This interaction slows down the platelets enabling them to interact with the exposed collagen via GPVI and platelet activation to ensue [57–59]. Binding of collagen to GPVI promotes an intracellular signalling cascade involving tyrosine kinase-mediated (e.g., Syk) activation of PLC*γ*2. The degradation of PI(4,5)P_2_ by PLC*γ*2 into diacylglycerol (DAG) and inositol 1,4,5-trisphosphate (Ins(1,4,5)P_3_, also known as IP_3_) induces indirect activation of protein kinase C (PKC) [59].

Platelet activation induces shape change and modulation of integrin *α*_IIb_*β*_3_ affinity to allow the formation of a platelet plug with fibrinogen used as a bridging molecule to surrounding platelets [5, 6]. Integrin activation is critical for a successful aggregation response. In resting platelets, integrin *α*_IIb_*β*_3_ is in a low affinity state but a conformational change during platelet activation enables high-affinity binding of ligands. PKC activation has a key role in modulating integrin *α*_IIb_*β*_3_ affinity [59, 60].

Furthermore, activation of platelets leads to degranulation and the secretion of adenosine diphosphate (ADP) and the synthesis and release of thromboxane A_2_ (TxA_2_), resulting in the activation of more platelets and recruitment of them to the thrombus [57–59]. Moreover, prothrombin is cleaved into thrombin following interactions involving tissue factor, factor VIIa, and factor Xa on the activated platelet surface. Thrombin is able to activate platelets through a cleavage of a region in the extracellular domains of protease-activated receptors (PARs) 1 and 4. Together, these agonists activate more circulating platelets and thus stimulate the formation of a platelet plug to seal the damaged region [5–7].

### 3.2. TLR4 Expression in Platelets

The presence of TLR4 on platelets is not disputed, and it was first identified on mouse and human platelets using flow cytometry by Andonegui et al. [36]. In addition, the same research group demonstrated that TLR4 displays functional effects in platelets. Furthermore, the discovery was backed up independently by Cognasse et al. in the same year, also through flow cytometry-based experiments [41]. Other research groups have also confirmed the presence of TLR4 on platelets through immunoblot analysis [42, 61, 62]. The amount of TLR4 expressed on the surface of platelets is variable, and an intracellular pool has also been identified [30, 41, 42].

**Figure 4:**
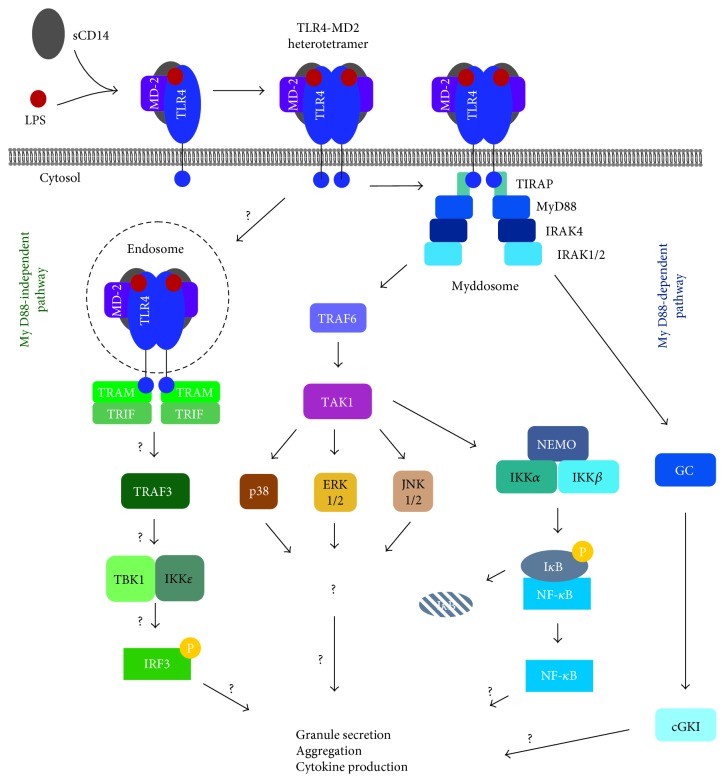
Summary of intracellular TLR4 signalling in platelets. Although the individual steps of the MyD88-dependent pathway have been observed, signalling in its entirety downstream of TLR4 has not been confirmed. Similar to nucleated cells, the proteins required for signalling through MyD88-dependent and -independent pathways are present in platelets but it is currently unclear how they mediate their effects. Signalling downstream of MyD88 can also be mediated by cGKI. The presences of TLR4, MyD88, IRAK1, TRAF6, TAK1, JNK, MAPK, I*κ*B*α*, NF-*κ*B, TRIF, TRAF3, TBK1, IKK*ε*, and IRF3 have all been confirmed by immunoblot analysis [2, 25]. Question marks (?) denote aspects of the pathway that have not been confirmed.

A big difference in TLR4 signalling between platelets and nucleated cells is that although platelets contain the intracellular signalling proteins required for TLR signalling (Figure 4), they do not have all of the necessary extracellular components (e.g., CD14) [2, 63, 64]. Membrane-bound CD14 is absent in platelets; however, this problem is overcome by high levels of soluble CD14 in the plasma [14, 30, 63, 65]. This may prevent “priming” of platelets at low concentrations of LPS whilst responses at higher concentrations are not affected. Moreover, the requirement for higher concentrations of LPS could prevent NET formation in response to minor bacterial infections, thus protecting against unwarranted endothelial damage [22]. Furthermore, the absence of membrane-bound CD14 may also have an impact on MyD88-independent signalling which requires CD14 for the endocytosis of TLR4 and LPS [55]. The loss of CD14 caused by “washing” platelets appears to reduce the magnitude of the response to LPS although a response is still present [63, 66, 67].

### 3.3. TLR4 Activity in Platelets

A strong piece of evidence for TLR4 activity in platelets comes from experiments conducted by Clark et al. They demonstrated that high concentrations of LPS led to an interaction between platelets and neutrophils that stimulated the formation of NETs [22]. The researchers also linked this activity to sepsis, a disease that is commonly associated with platelet TLR4 [19]. This was achieved by determining the production of NETs in the blood samples of sepsis patients [22]. It is unclear whether it was the LPS in the blood or another substance that stimulated this response as certain proteins that may stimulate platelets in a TLR4-dependent manner are also released into the blood during sepsis, for example, HMGB1 [22, 32]. Similarly, further evidence for the role of platelet TLR4 is provided by the observation that the levels of soluble cluster of differentiation 40 ligand (sCD40L) are raised following treatment of platelets with LPS [63, 66–69]. This is important because platelet *α*-granules are the largest source of sCD40L, and CD40L is involved in inflammatory responses elucidated by the endothelium and immune cells [1, 68–70]. Increases in sCD40L levels have been suggested to directly involve TLR4 [69].

Many attempts have been made to characterise the responses of platelets to LPS and other TLR4 agonists although there have been conflicting results. Evidence from different studies agree that exposure of platelets to LPS stimulates the release of tumour necrosis factor- (TNF-) *α*, a molecule that is produced downstream of the MyD88-dependent pathway in nucleated cells [42, 50, 71]. Although platelets lack genomic DNA, they still contain mRNA transcripts that can be processed and spliced following stimulation of platelets by LPS or thrombin [63, 72]. Transcripts that are affected include IL-1*β* (a proinflammatory cytokine) and cyclooxygenase-2 (produces a platelet agonist, TxA_2_) [63]. In addition, IL-1*β* mRNA has been shown to be spliced in platelets in a TLR4-dependent manner with JNK and protein kinase B (PKB) (found downstream of the MyD88-dependent pathway) activity increasing during splicing [65]. Furthermore, splicing of IL-1*β* was diminished in the presence of JNK or PKB inhibitors. However, the mechanism of action has not yet been elucidated [65]. Platelet shape change as a result of actin filament formation has also been suggested [63]. A comprehensive examination of cytokine release from platelets after treatment with LPS was conducted by Cognasse et al. [30]. They demonstrated that the expression of CD63 and release of sCD40L and platelet-activating factor 4 (PAF4) were increased; release of regulated upon activation, normally T-expressed, and presumably secreted (RANTES), angiogenin and platelet-derived growth factor- (PDGF-) AB were decreased (along with TLR4 expression); meanwhile, there was no change in the expression of soluble P-selectin, epidermal growth factor (EGF), transforming growth factor *β* (TGF*β*), or IL-8 [30]. Upregulation of P-selectin following LPS exposure is controversial with evidence both for [32, 61, 63] and against [26, 30, 66].

### 3.4. The Role of MyD88 in Platelets

It is unclear whether the traditional TLR4 pathways are responsible for all the effects mediated by TLR4 ligands on platelet function. MyD88^−/−^ mouse platelets have been used to demonstrate that this protein is necessary for the effects of LPS in enhancing aggregation and granule secretion in platelets. Some effects downstream of MyD88 have also been shown to be mediated by the cyclic guanosine monophosphate- (cGMP-) mediated signalling pathway [61].

In contrast, one research study demonstrates that there is virtually no role for MyD88 in modulating platelet function during Gram-negative (*Klebsiella pneumoniae*) bacterial infection [71]. Differences in responses were observed in systemic MyD88^−/−^mice compared to the controls; however, these differences could not be recovered by transfusing wild-type platelets into the MyD88^−/−^ mice. Furthermore, some changes in the secretion of TNF*α* and monocyte chemoattractant protein-1 (MCP-1) were observed that could be the result of deletion of platelet MyD88, thus suggesting that signalling to NF-*κ*B is still intact and functioning [71].

The results of this study are somewhat limited for several reasons. For example, the observed effects were not shown to be mediated by TLR4 as competitive antagonists, blocking antibodies for TLR4, and platelets derived from TLR4-deficient mice were not used in their experimental settings. Furthermore, this study did not use pure LPS (or other potential TLR4 ligands), but rather whole *Klebsiella pneumoniae* bacteria, which means that other bacterial components or exotoxins may have been able to influence cellular activities. More specifically, there was no investigation into the success of the platelet transfusions as the recipient mice were not depleted of their platelets and transfused platelets may have been sequestered in organs such as the lungs and spleen. The possibility of adaptive mechanisms in the MyD88-deficient mice was not investigated either; nor was an alternative signalling pathway suggested. Nevertheless, this study highlights the necessity for further research in order to confirm the significance of MyD88 in TLR4-mediated signalling in platelets.

### 3.5. Priming Platelets

There is evidence suggesting that LPS (and therefore TLR4-mediated signalling) has a “priming” role in platelets. LPS on its own is unable to induce aggregation in washed platelets, but it can potentiate agonist-induced aggregation responses. This was elucidated through the use of classical agonists such as collagen and thrombin [14, 26, 61]. Despite washed platelets being used, sCD14 was still detectable on platelets via flow cytometry [61]. Similar results have been obtained with platelet-rich plasma (PRP) using agonists such as adenosine diphosphate (ADP) [63]. The response was mediated by TLR4 as demonstrated through the use of TLR4^−/−^ mouse platelets [61]. An intriguing observation from this was that the different bacterial strains of LPS tested had different potencies [61]. This has also been observed with the LPS from *Rhodobacter sphaeroides* demonstrating its ability to act as a competitive TLR4 antagonist [63]. This priming behaviour in platelets is also supported by studies using NF-*κ*B and IKK*β* inhibitors [26, 73, 74].

The identification of TLR4:MyD88 coupling to the cGMP-dependent pathway is important as this pathway stimulates platelet aggregation from a subthreshold concentration of an agonist (0.02 U/mL of thrombin) [75]. Thus, there is a precedent for TLR4 to have a priming role in platelet aggregation. The response to cGMP-analogues was biphasic with an initial stimulatory response followed by an inhibitory response [75]. An interesting point to consider is that whilst cGMP-dependent kinase I (cGKI) inhibition affected aggregation and secretion to low agonist concentrations (excluding ADP), there was no effect on calcium mobilisation [76] and TLR4 is also incapable of modulating calcium mobilisation [77]. cGKI has been proposed to be involved positively in the G_i_-pathway, and so activation of cGKI could help amplify platelet responses in a similar manner to the P2Y_12_ receptor [76].

### 3.6. NF-*κ*B in Platelets

Given that platelets lack a nucleus, it may appear that the presence of a signal transduction pathway that culminates in transcription factor activation would have no role in platelet function. This initially prompted some researchers to claim that TLR4 and its downstream signalling proteins in platelets were relics left over from their formation by megakaryocytes. Furthermore, certain experiments concluded that there were no responses mediated by TLR4 with specific bacterial species, lending support to this argument [78]. Other concerns arose from different LPS chemotypes derived from diverse bacterial species having diverse potencies when it comes to elucidating a response [61, 63, 66, 79]. NF-*κ*B, however, appears to have a role in platelet function, suggesting a nongenomic role, especially when the ability of NF-*κ*B inhibitors to reduce the proaggregatory effects of TLR4 is considered [26, 80].

Notably, IKK*β* is involved in the phosphorylation of soluble N-ethylmaleimide-sensitive factor attachment protein receptors (SNAREs), particularly synaptosomal-associated protein 23 (SNAP23), and thus, IKK*β* has an important role in granular secretion [73]. Phosphorylation of SNAREs is known to occur downstream of PKC when thrombin is used as an agonist [73]. This is relevant because IKK*β* is found downstream of both this classical agonist pathway and the MyD88-dependent pathway, suggesting a mechanism by which TLR4 activation could lead to the secretion of granules that has been shown in some studies [32, 51, 73]. Further investigations have revealed that IKK*β* activity occurs downstream of TAK1, found in the MyD88-dependent pathway [25]. This evidence points towards the ability of the MyD88-dependent pathway to promote SNARE complex formation and may explain some of the “priming” activity induced by TLR4 ligands. However, it is unclear whether IKK*β* directly phosphorylates SNAP23 or whether it occurs due to the activation of NF-*κ*B. It has been shown that NF-*κ*B activity is involved in modulating dense and *α*-granule secretion upon activation with low agonist doses by using inhibitors of I*κ*B*α* phosphorylation and ubiquitination (to indirectly inhibit NF-*κ*B activity) [74, 80]. Moreover, NF-*κ*B inhibition decreases binding of platelets to fibrinogen [80]. This suggests that NF-*κ*B is responsible for modulating secretion in this case although one of the inhibitors used is likely to directly inhibit IKK*β*. Inhibition of aggregation has also been seen to be mediated by NF-*κ*B inhibitors downstream of TLR4, suggesting that TLR4 and NF-*κ*B activity is connected in platelets [26].

### 3.7. Other Ligands

Although LPS has been the predominant ligand mentioned in this review, other ligands have also been suggested to bind to TLR4; however, this area is highly controversial [9, 67, 81, 82]. HMGB1 is one such possible ligand and has been shown to have effects in platelets in an autocrine and paracrine manner [32]. With a presence in the plasma and on NETs, the DNA-binding protein released from dead/dying cells or activated immune cells has opportunities to interact with platelets in many conditions, for example, sepsis [22, 32, 83, 84]. HMGB1 has been reported to elicit similar responses in platelets compared to LPS, including the priming effects. These effects were also shown to involve TLR4, MyD88, and cGKI although there is not yet clear evidence indicating exactly how these proteins relate. ERK was another protein that had a change in its activity as a result of treatment with HMGB1 dependent on the presence of TLR4 [32]. HMGB1 has also been shown to have a role in tumour metastasis in a mechanism involving TLR4 [84]. Platelets are known to aid in cancer metastasis by forming protective thrombi around metastasising cells [85], and subsequent experiments by Yu et al. demonstrated that deletion of TLR4 in mice led to fewer metastatic tumours [84]. However, evidence from nucleated cells exists implying that HMGB1 acts solely as a TLR ligand-binding protein (e.g., LPS) and potentiates signalling through TLRs (alarmin effect) [86, 87]. Thus, the effects observed in the studies might be due to the binding of HMGB1-LPS colligation to TLR4 [87]. Moreover, recent studies have shown that, instead of direct binding to TLR4, HMGB1 directly exerts effects (such as activation of NF-*κ*B and MAPKs) on cells through binding to the receptor for advanced glycosylation end-products (RAGE) [88].

Another ligand that has been suggested to alter platelet activity in a TLR4-dependent manner is cellular fibronectin [9]. It has been shown that cellular fibronectin can modify platelet activity in a similar manner to LPS by potentiating aggregation induced by low doses of thrombin and increasing phosphorylation of NF-*κ*B and IKK*α*/*β* [9]. Furthermore, it was shown that the presence of TLR4 in mouse platelets significantly increased thrombus growth when treated with cellular fibronectin [9]. These findings suggest a possible effect of cellular fibronectin that may be mediated in a TLR4-dependent manner.

Histones have also been proposed to be ligands for TLR4 and are found in the blood during sepsis following release from neutrophils or necrotic cells [89–92]. They are important for the organisation of DNA in nucleated cells and, like HMGB1, appear in NETs [93]. Histones (especially H4) have interactions in the blood, and they have been reported to have a role in chemokine production in whole blood, platelet aggregation, and also thrombocytopenia in mice [89, 93]. However, these studies concluded that it was monocytes, and not platelets, that were responsible for the TLR4-dependent production of cytokines (even though histone H4 did associate with platelets) whereas the impact of TLR4 on histone-induced aggregation and thrombocytopenia was not examined [89, 93]. In contrast, it has been shown that histones can stimulate P-selectin exposure and thrombin generation on platelets in a TLR2- and TLR4-dependent manner [81].

Additionally, HSP60, a cell-stress marker, has been proposed to trigger TLR4-mediated signalling in a vascular smooth muscle cell line, with implications in atherosclerosis. However, the effects of this protein have not been tested on platelets despite an increased expression of HSP60 on endothelial cells in sheer stress environments [94, 95]. Serum amyloid A (SAA) is a potential ligand for TLR4 that is released, primarily from the liver, during an inflammatory response [96, 97]. Platelets have been shown to adhere to SAA in an integrin *α*_IIb_*β*_3_-dependent manner; however, it has not been determined whether or not this integrin is solely responsible for this behaviour as the research was conducted before the discovery of TLR4 on platelets [97]. Further research is required to determine whether these proposed ligands are having an effect due to direct binding to TLR4 or if it is the result of a more complex interaction, as has been suggested for HMGB1 [87].

## 4. TLR4 Signalling in Platelets: What Is Still to Discover?

Although many studies have linked TLR4 activity in platelets to immune responses, there have not been many studies to explore the signalling pathways downstream of TLR4 or MyD88 [26, 32, 61]. This is of particular interest as this receptor, with so many potential ligands and possible functions, operates through a pathway that classically results in gene transcription, but this end result is not achievable due to the lack of a nucleus in platelets. Moreover, the presence of all the signalling proteins in the pathways has been confirmed [2, 25] but whether the entirety of each pathway is functional, in platelets, has not been elucidated. Currently, individual steps of the MyD88-dependent pathway have been seen but not tied together downstream of TLR4. The MyD88-independent pathway in platelets also lacks considerable amounts of detail, including study of its activity. Furthermore, platelet TLR4 expression levels have been linked to more severe disease states in inflammatory responses [8, 9, 81, 84, 98–101]. This obviously makes TLR4 an interesting receptor to target for the prevention and/or treatment of cardiovascular diseases. However, it is challenging due to the important contribution of TLR4 to innate immunity. Determination of the effector proteins involved and their responses may lead to the discovery of novel pathways downstream of TLRs and present TLR4 as a novel therapeutic target for the treatment of cardiovascular diseases and other pathological settings such as inflammatory disease.

## Abbreviations

ADP: Adenosine diphosphate
AP-1: Activator protein 1
CD63: Cluster of differentiation 63
cGKI: cGMP-dependent kinase I
cGMP: Cyclic guanosine monophosphate
cIAP: Cellular inhibitor of apoptosis
DAG: Diacylglycerol
DAMPs: Damage-associated molecular patterns
DD: Death domain
DIC: Disseminated intravascular coagulation
EGF: Epidermal growth factor
ERK: Extracellular signal-regulated kinase
GP: Glycoprotein
gp96: Heat shock protein 90 kDa *β* member 1
GPI: Glycosylphosphatidylinositol
HSP60: Heat shock protein 60
HMGB1: High-mobility group box 1
HSP: Heat shock protein
I*κ*B: Inhibitor of NF-*κ*B
IKK: I*κ*B kinase
IL: Interleukin
Ins(1,4,5)P_3:_: Inositol 1,4,5-trisphosphate
IRAK: IL-1 receptor-associated kinase
IRF3: Interferon regulatory factor 3
JNK: c-Jun N-terminal kinase
LBP: Lipopolysaccharide-binding protein
LPS: Lipopolysaccharide
LRR: Leucine-rich repeat
Mal: MyD88 adaptor-like
MAPK: Mitogen-activated protein kinases
MCP-1: Monocyte chemoattractant protein-1
MD-2: Myeloid differentiation factor-2
MyD88: Myeloid differentiation factor 88
NEMO: NF-*κ*B essential modulator
NET: Neutrophil extracellular trap
NF-*κ*B: Nuclear factor of *κ*-light-polypeptide-gene-enhancer in B cells
PAF4: Platelet-activating factor 4
PAR: Protease-activated receptor
PDGF-AB: Platelet-derived growth factor-AB
PI(4,5)P_2:_: Phosphatidylinositol-4,5-bisphosphate
PKB: Protein kinase B
PKC: Protein kinase C
PLC*γ*2: Phospholipase C*γ*2
PRAT4A: Protein associated with TLR4
PRP: Platelet-rich plasma
RAGE: Receptor for advanced glycation end-products
RANTES: Regulated upon activation, normally T-cell expressed, and presumably secreted
SAA: Serum amyloid A
(s)CD14: (Soluble) cluster of differentiation 14
sCD40L: Soluble cluster of differentiation 40 ligand
SNAP23: Synaptosomal-associated protein 23
SNARE: Soluble N-ethylmaleimide-sensitive factor attachment protein receptor
Syk: Spleen-associated tyrosine kinase
TAK1: TRAF-activated kinase 1
TBK1: TRAF family member-associated NF-*κ*B activator- (TANK-) binding kinase 1
TGF*β*: Transforming growth factor *β*
TIR: Toll/IL-1 receptor
TIRAP: TIR domain-containing adaptor protein
TLR: Toll-like receptor
TNF: Tumour necrosis factor
TRAF: TNF receptor-associated factor 3
TRAM: TRIF-related adaptor molecule
TRIF: TIR domain-containing adaptor-inducing interferon-*β*
TxA_2:_: Thromboxane A_2_
vWF: von Willebrand factor.
